# Effect of Exposure to Environmental Cycling on the Thermal Conductivity of Expanded Polystyrene

**DOI:** 10.3390/ma15196921

**Published:** 2022-10-06

**Authors:** Sergiu George Petre, Dorina Nicolina Isopescu, Marian Pruteanu, Alexandra Cojocaru

**Affiliations:** Department of Civil and Industrial Engineering, “Gheorghe Asachi” Technical University of Iasi-Romania, Blvd. Mangeron, No. 1, 700050 Iasi, Romania

**Keywords:** building energy performance, thermal resistance, expanded polystyrene freeze–thaw cycles, environmental cycling

## Abstract

The global effort to reduce energy consumption generated by buildings involves the increasing use of thermal insulation materials, with expanded polystyrene representing one of the most used materials to this end. The thermal performance of expanded polystyrene has been extensively studied; however, information on the effect of exposure to cyclic environmental conditions on its thermal performance is limited. Therefore, we conducted such a study, which is presented in this article. In the first stage, samples were subjected to 28 days of preconditioning to accelerate the increase in moisture in the material. The second stage involved exposure to 40 freeze–thaw cycles of 12 h each. The weight and thermal conductivity of the samples were measured before and after exposure, and the compression behavior was also analyzed. The results show a decrease in the thermal performance of expanded polystyrene exposed to cyclic environmental conditions, as demonstrated by an increase in the water content of the material under the same measurement conditions and an increase of 5.06% in the average thermal conductivity. The influence of this increase on the thermal performance of commonly used wall construction elements was also been studied and presented in this article. A decrease of 7.59% in the compressive stress of the material at 10% strain was also demonstrated.

## 1. Introduction

Currently, the effects of global warming do not affect all the inhabitants of the planet in the same way; however, as time passes, more people will be affected by high temperatures, floods, drought, forest fires, and rising sea and ocean levels [1,2]. The building sector accounts for approximately 40% of the total energy consumed in the European Union, and 68% of the total energy consumption in residential buildings is generated by space heating [2]. Considering the high level of energy consumption generated by buildings [3,4,5], construction specialists must pay special attention to insulation materials used in the realization of new construction and the modernization of existing buildings.

Among the conventional thermal insulation materials used in the construction sector, in recent years, expanded polystyrene has experienced explosive growth [6]. Due to its high thermal performance and low cost, expanded polystyrene held a market share of 27% in the European Union in 2015, the second largest after that of glass wool, which occupied a market share of 36% [7]. As one of the most widely used thermal insulation materials in construction and considering the forecasted increased level of use, extensive knowledge of the behavior of expanded polystyrene from all points of view is essential.

The technical characteristics and behavior of expanded polystyrene in various situations have been studied intensively, so the literature is replete with research on the mechanical characteristics [8,9,10], fire behavior [11,12,13], and even the toxicity and environmental impact [14,15,16] of this material.

Considering that expanded polystyrene is widely used as a thermal insulating material in construction, its thermal performance has also been studied intensively. Generally, such studies of this kind investigate the variation of thermal conductivity of the material under varying loads. The literature includes studies showing variations in the thermal conductivity of expanded polystyrene depending on temperature [17,18,19], humidity [20], the combined effect of temperature and humidity [21,22], and density [23,24,25].

Unlike widely published research on the influence of temperature and humidity on the thermal performance of materials, the influence of exposure to outdoor environmental cycles on the thermal performance of thermal insulating materials is much less studied. A limited number of studies have been conducted on the degradation of thermal performance of materials due to exposure to freeze–thaw conditions, in particular for extruded polystyrene [26,27].

In this article, we present a study to assess the effect of exposure to outdoor environmental cycles on the thermal performance of expanded polystyrene. The experimental study consists in determining the influence of the cyclicality of environmental conditions on expanded polystyrene by identifying changes in the thermal conductivity and in the mechanical properties of the material. The results show a reduction in the thermal performance of expanded polystyrene exposed to cyclic environmental conditions, as well as an increase in thermal conductivity. A reduction in the compressive strength of the material was also observed.

To quantitatively determine how an increase in the thermal conductivity of polystyrene influences the thermal performance of its component elements, a study was conducted analyzing the heat transfer by conduction for nine building elements. Calculations were performed considering the thermal conductivity of polystyrene before and after exposure, with the results of the study presented in this article.

## 2. Materials and Methods

### 2.1. Materials and Apparatus

The material selected for the experimental study were an expanded polystyrene board (EPS 80), which was produced according to EN 13163:2012 [28]. The density of the material is 14.5 kg/m^3^, the compressive stress at 10% strain is 80 kPa, and the declared thermal conductivity is 0.038 W/mK. The technical sheet of the product under consideration shows that the frost–thaw resistance has not been determined.

**Figure 1:**
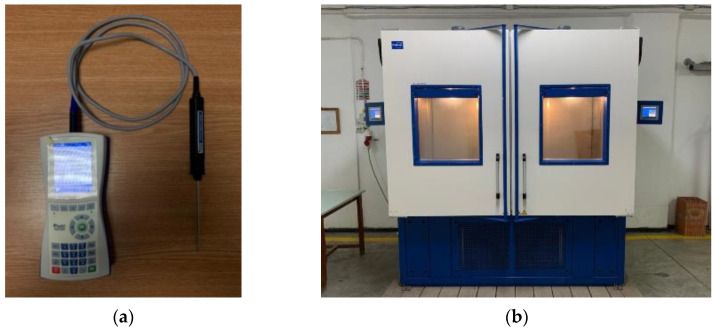
(**a**) Heat transfer analyzer; (**b**) twin test climate chamber.

The test apparatus includes a high-precision electronic balance, a system for measurement of heat transfer properties of materials (Isomet 2114 Applied Precision with a measurement accuracy of the thermal conductivity value, for samples with thermal conductivity values between 0.015 and 0.70 W/mK of 5% of the measured value +0.001 W/mK) (Figure 1a) and a twin test climate chamber (Feutron Klimasimulation GmbH), which was used for freeze–thaw cycle simulations (Figure 1b). A computer-controlled electrohydraulic testing machine was used to determine the compression behavior (WAW-600E Jinan Testing Equipment IE Corporation, Jinan, China) of the tested material. Other instruments were also used for cutting and measurement of the samples.

### 2.2. Experimental Procedure

**Figure 2:**
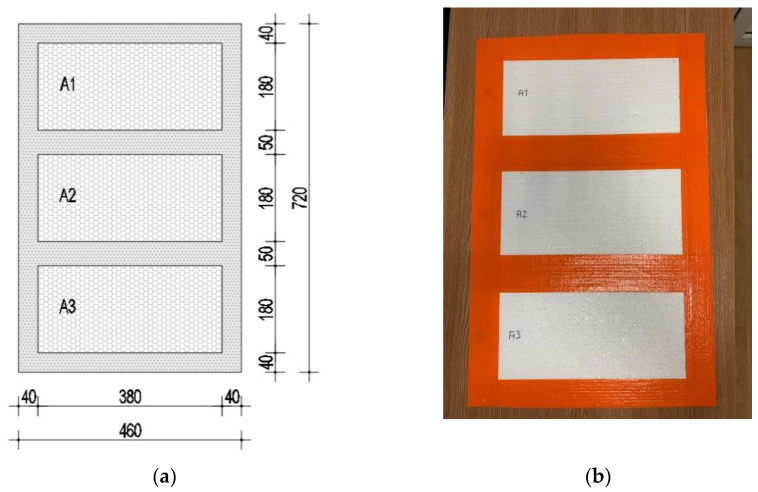
(**a**) Drawing of the panel; (**b**) final panel for testing. (Unit: mm).

Three samples measuring 380 mm × 180 mm × 30 mm (length × width × thickness) made of expanded polystyrene were tested. The samples were mounted in a frame made of extruded polystyrene with the same thickness as the analyzed samples. A drawing of the panel is shown in Figure 2a. In order to ensure that mass transfer was realized through the analyzed samples and not through the joints between the two materials, they were sealed with vapor-proof adhesive tape. Figure 2b shows the sealed end panel.

**Figure 3:**
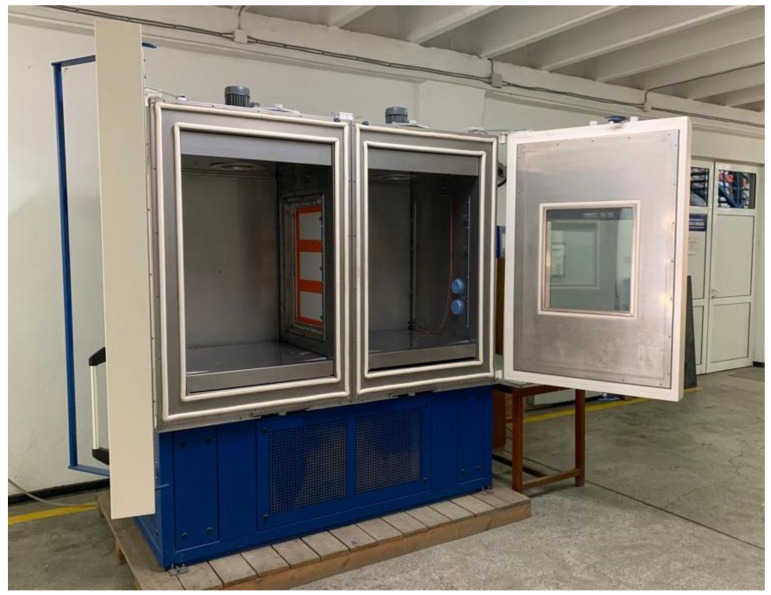
Test panel mounted as a partition.

The obtained panel was mounted in the frame for the partition of the climate chambers, a wall that separates the warm room from the cold room. Figure 3 shows the test panel mounted as a partition.

Tests were conducted according to the ASTM C1512-10 standard (2020) [29] in two stages: the preconditioning stage and exposure to freeze–thaw cycles. 

In the first stage, to increase the rate at which the water content of the analyzed material increased, the samples were subjected to a constant difference in temperature and relative humidity. Thus, a temperature of 24 °C and a relative humidity of 90% were set for 28 days in the warm room, and in the cold room a temperature of −15 °C was set, without imposing a value for relative humidity.

The second stage involved exposure of the samples to 40 12 h freeze–thaw cycles. A complete cycle consists of two equal periods of exposure to warm and cold. Throughout the second stage, a temperature of 24 °C and a relative humidity of 90% were set in the warm room. In the cold room, the temperature was set at 15 °C during warm exposure and to −15 °C during cold exposure. In the cold room, no relative humidity values were imposed according to the standard requirements.

To quantitatively establish the thermal performance of the test samples, thermal conductivity measurements were taken before and after the test. Before the thermal conductivity measurements of the samples, they were conditioned at a temperature of 23 °C and a relative humidity of 50% for 48 h.

According to standardized prescriptions, compressive strength measurements were performed for samples exposed and unexposed to freeze–thaw cycles with the aim of determining the extent to which exposure of expanded polystyrene to environmental conditions alters its compression behavior, mainly the compressive stresses in response to 10% strain.

## 3. Results

Three thermal conductivity measurements were taken for each sample before and after exposure of samples to freeze–thaw cycles, and the weight of the samples was also measured. The measurements were conducted after the samples went through a conditioning cycle. The results of the measurement before the exposure are presented in Table 1.

The results of measurements of the samples obtained after exposure to 40 freeze–thaw cycles are presented in Table 2.

Freeze–thaw cycles can cause degradation in the structure of a porous material, such as expanded polystyrene. This progressive damage affects the resistance to various demands, with the material showing lower values of properties as freeze–thaw cycles increase. This effect can be attributed to the phenomenon of aging of the material. The standard [29] recommends evaluation of the compressive resistance of the material. According to standardized recommendations, tests were performed, with the results obtained for exposed samples (A1, A2, and A3) presented in Table 3. The results of the test for unexposed samples (B1, B2, and B3) are presented in Table 3 to highlight the reduction in compressive stresses and to allow for quick comparison.

## 4. Discussion

**Figure 4:**
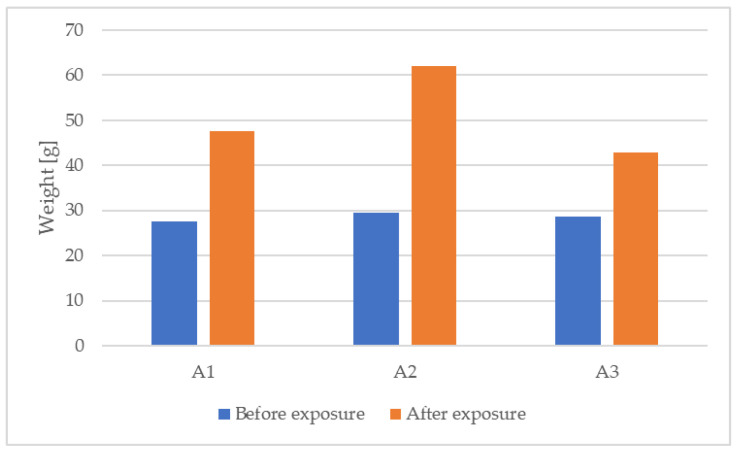
Graphic representation of weight variation.

An increase in the weight of the samples measured under the same conditions following conditioning was observed after exposure to the freeze–thaw cycles. A summary of the weight measurements before and after sample exposure is shown in Table 4, and a graphic representation of the variation is presented in Figure 4.

The increase in the weight of the samples was, on average, 77.56%, indicating an increase in the water content for the same conditions. Considering that a higher water content causes the thermal conductivity of the material to increase, it can be said that the thermal performance of the material is affected by exposure to environmental cycling.

**Figure 5:**
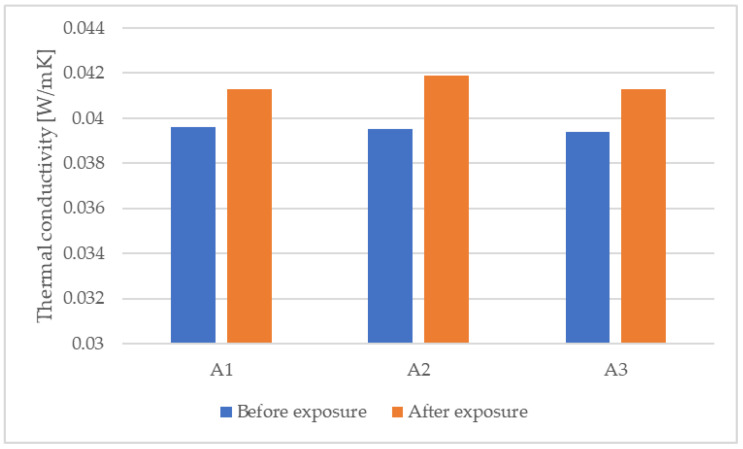
Graphic representation of the variation of the thermal conductivity.

The decrease in thermal performance of expanded polystyrene samples is also shown by the results of thermal conductivity measurements. A summary of thermal conductivity measurements before and after sample exposure is shown in Table 5, and a graphic representation of the variation is presented in Figure 5.

The increase in thermal conductivity of the analyzed material was, on average, 5.06%, with a direct impact on the thermal performance of the building elements. To quantitatively establish this influence, calculations of heat loss by conduction were performed for nine elements according to Equation (1).

(1) $$Q=\frac{S\cdot \left(T_{si}-T_{se}\right)\cdot \tau }{R},$$

where *Q* represents the amount of heat transmitted by conduction (Wh); *S* represents the area of the surface of the element through which heat transfer occurs perpendicular to the direction of heat propagation (m^2^); *T_si_*, and *T_se_* represent the temperatures of the inner and outer surfaces of the element, respectively (°C and K, respectively); *τ* represents the time (h); and *R* represents the unidirectional thermal resistance of the element (m^2^K/W).

**Figure 6:**
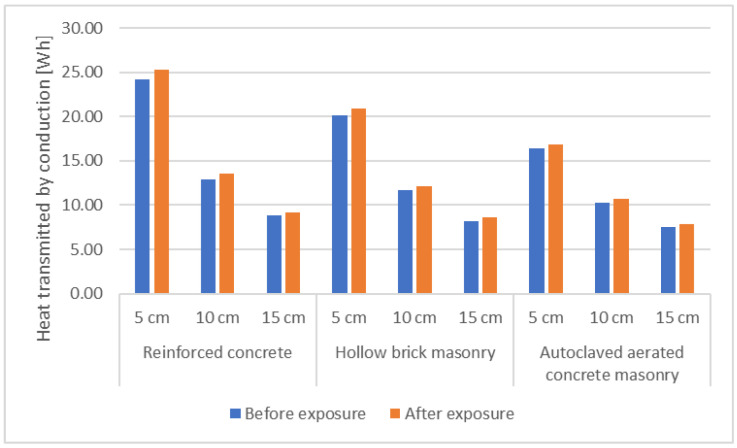
Graphic representation of the variation of the heat transmitted by conduction.

Three types of wall elements were considered: with the structural layer made of reinforced concrete, with the structural layer of hollow brick masonry, and with the structural layer made of autoclaved aerated concrete masonry. For each element, the thermal insulation layer was made of expanded polystyrene with a thickness of 5, 10, and 15 cm, and the thermal conductivity measured before and after the exposure of the samples was used in the calculations. The heat losses by conduction were calculated for each of the elements, considering that transfer occurs through an element with an area of 1 m^2^ for one hour with a temperature difference of 35 °C. A summary of the estimated results is shown in Table 6, with a graphic representation presented in Figure 6.

The results show that the impact of increased conductivity from exposure of samples to freeze–thaw cycles on the thermal performance of an element is greater with increased thickness of the element’s thermal insulation layer and with lower thermal performance of the other component layers. On average, the heat transfer by conduction increased by 4.12% for the elements for which thermal conductivity obtained after exposure of the samples was used in calculations.

The decrease in the mechanical performance of expanded polystyrene samples is also demonstrated by the results of compression behavior determinations. These results show a decrease of 7.59% in compression stress at 10% strain of samples exposed to freeze–thaw cycles compared to unexposed samples.

## 5. Conclusions

Expanded polystyrene is a low-cost thermal insulator material with a good thermal performance; therefore, it is a preferred material that is increasingly used to improve the thermal performance of buildings and to decrease consumption generated by their use.

In this article, we present a study on the effect of exposure to cyclic environmental conditions on the thermal performance of expanded polystyrene. To quantify the effects, changes in weight, mechanical properties, and thermal conductivity were analyzed before and after exposure of the samples to freeze–thaw cycles.

The results, show that for the same type of conditioning, the weight of the samples after exposure to freeze–thaw cycles increased, on average, by 77.56%, indicating an increase in the water content of the material.

The thermal conductivity of the test samples increased, on average by, 5.06%, indicating a degradation of the thermal performance of the material following exposure to cyclic environmental conditions. The impact on the thermal performance of some wall building elements was analyzed, and an increase in the amount of heat transmitted by conduction of between 3.58% and 4.64% was observed, depending on the type of element.

Mechanical degradation was also observed, with decrease in compression stress of 7.59% at 10% strain.

Improved knowledge of the behavior of expanded polystyrene in the event of exposure to cyclic environmental conditions is necessary, and studies considering varying thicknesses and qualities of test materials should be conducted in the future.

## Figures and Tables

**Table 1:** Initial measurement values.

Sample	Weight (g)	Thermal Conductivity (W/mK)			
		Measurement 1	Measurement 2	Measurement 3	Mean Value
A1	27.62	0.0395	0.0396	0.0396	0.0396
A2	29.41	0.0394	0.0396	0.0396	0.0395
A3	28.60	0.0393	0.0395	0.0394	0.0394

**Table 2:** Final measurement values.

Sample	Weight (g)	Thermal Conductivity (W/mK)			
		Measurement 1	Measurement 2	Measurement 3	Mean Value
A1	47.50	0.0411	0.0413	0.0414	0.0413
A2	62.09	0.0417	0.0420	0.0420	0.0419
A3	42.78	0.0412	0.0414	0.0413	0.0413

**Table 3:** Compressive stress at 10% strain for exposed and unexposed samples.

Unexposed		Exposed	
Sample	Compressive Stress at 10% (kPa)	Sample	Compressive Stress at 10% (kPa)
B1	76.00	A1	71.00
B2	76.00	A2	68.00
B3	75.00	A3	72.00
**Mean value**	**75.67**	**Mean value**	**70.33**

**Table 4:** Summary of weight measurement values before and after environmental exposure.

Sample	Weight (g)		Percentage Difference (%)
	Before Exposure	After Exposure	
A1	27.62	47.50	+71.98
A2	29.41	62.09	+111.12
A3	28.60	42.78	+49.58
**Mean value**	**28.54**	**50.79**	**+77.56**

**Table 5:** Summary of thermal conductivity measurement values before and after environmental exposure.

Sample	Thermal Conductivity (W/mK)		Percentage Difference (%)
	Before Exposure	After Exposure	
A1	0.0396	0.0413	+4.29
A2	0.0395	0.0419	+6.08
A3	0.0394	0.0413	+4.82
**Mean value**	**0.0395**	**0.0415**	**+5.06**

**Table 6:** Summary of heat transmitted by conduction before and after environmental exposure.

Element		Heat Transmitted by Conduction (Wh)		Percentage Difference (%)
		Before Exposure	After Exposure	
Reinforced concrete	+5 cm	24.19	25.25	+4.40
	+10 cm	12.90	13.51	+4.71
	+15 cm	8.80	9.22	+4.82
**Mean value**				**+4.64**
Hollow brick masonry	+5 cm	20.18	20.91	+3.65
	+10 cm	11.67	12.16	+4.24
	+15 cm	8.20	8.57	+4.48
**Mean value**				**+4.12**
Autoclaved aerated concrete masonry	+5 cm	16.38	16.86	+2.94
	+10 cm	10.29	10.67	+3.72
	+15 cm	7.50	7.80	+4.08
**Mean value**				**+3.58**

## Data Availability

Not applicable.
